# The Effect of Liquid Phase Concentration on the Setting Time and Compressive Strength of Hydroxyapatite/Bioglass Composite Cement

**DOI:** 10.3390/nano11102576

**Published:** 2021-09-30

**Authors:** Shamsi Ebrahimi, Coswald Stephen Sipaut

**Affiliations:** Faculty of Engineering, Universiti Malaysia Sabah, UMS Road, Kota Kinabalu 88400, Sabah, Malaysia; ebrahimi_shamsi63@yahoo.com

**Keywords:** scaffolds, composite, setting time, compressive strength

## Abstract

Composite scaffolds of hydroxyapatite (HAp) nanoparticles and bioactive glass (BG) have been applied as appropriate materials for bone tissue engineering. In this study, hydroxyapatite/bioglass cement in different ratios was successfully fabricated. To prepare HAp and HAp/BG cement, synthesized HAp and HAp/BG powder were mixed in several ratios, using different concentrations of sodium hydrogen phosphate (SP) and water as the liquid phase. The liquid to powder ratio used was 0.4 mL/g. The results showed that setting time increased with BG content in the composite. The results also showed that with the addition of bioglass to the HAp structure, the density decreased and the porosity increased. It was also found that after immersion in simulated body fluid (SBF) solution, the compressive strength of the HAp and HAp/BG cements increased with BG concentration up to 30 wt.%. SEM results showed the formation of an apatite layer in all selected samples after immersion in SBF solution. At 30 wt.% BG, greater nucleation and growth of the apatite layer were observed, resulting in higher bioactivity than pure HAp and HAp/BG in other ratios.

## 1. Introduction

One of the main tasks in bone tissue engineering is the production of biocompatible and biodegradable scaffolds. Until now, scaffolds for making bone tissue and for angiogenesis have been fabricated using biocompatible and biodegradable materials and have also been designed for porousness. With these features, scaffolds can provide a good substrate for loading growth factors, drugs, genes, and cells. However, one of the main challenges in bone tissue engineering is to fabricate biocompatible and biodegradable scaffolds possessing an appropriate size and sufficient mechanical strength to improve the adhesion, growth, and differentiation of bone cells [1]. This challenge is the focus of the work described here. Calcium phosphate cement was developed in the early 1980s [2], and subsequent variations have been developed using a wide variety of formulations [3]. Calcium phosphate cement consists of two parts: powder and liquid, which yield a hardened solid after mixing using a specific weight ratio and time. The setting reaction product of calcium phosphate cement in most cases is HAp. Due to its formation at body temperature, HAp has a high specific surface and low crystallinity, and is very similar to the HAp in bone tissue [4,5]. The setting time of cement is directly related to its strength. The setting reaction is formed during the supersaturation and sedimentation stages, and the deposited HAp with interconnected micropores is structurally very similar to biological apatite. Considering the time constraints in surgery, the initial setting time and final setting time are crucial in calcium phosphate bone cement.

The initial setting time is when the cement is formed but does not deform naturally unless subject to an external force. The final setting time is when the cement can be touched without causing scratches or deformation [6]. The initial setting time should be adjusted to allow sufficient opportunity for a surgeon to fill and shape the cement. Between filling and complete stiffness, the cement should not be subject to any mechanical force which would cause scratching and negatively impact strength. Therefore, wound closure would be delayed until after the final setting time. For orthopedic applications, the initial setting time is 8 min, and the final setting time is 15 min [5].

The primary problem with calcium phosphate cement is obtaining cement with mechanical properties appropriate for bone tissue engineering. To date, extensive studies have been carried out to overcome the mechanical weakness of HAp for its wider application in the repair of hard damaged tissues, especially in areas under load. The addition of reinforcement to the structure of calcium phosphate ceramics and the improvement of processing and production methods have been considered the most important strategies to improve mechanical properties whilst maintaining biocompatibility [7,8,9,10]. One of the most important methods for improving calcium phosphate cement properties is by introducing reinforcement materials. The introduction of different amplifiers to calcium phosphate ceramic structures and improving building and processing methods are among the most important strategies for increasing the mechanical properties of apatite ceramics and maintaining their biocompatibility. Metal particles [11], biocompatible glasses [8,12], as well as neutral ceramic phases, such as alumina [13], zirconia [14], collagen [15,16], and titanic [17], are among the phases that are added to its structure to improve the mechanical properties of HAp. Additives such as bioglass (BG) are used as the second part of the cement composite to improve physical and mechanical properties, increase biological properties (such as the osteoinductive effect), and to reduce infectiousness [18]. Porous materials such as bioglass are materials that have controlled holes in their final structure. Porous materials have a large surface area and, as a result, have a high tendency to biosorption, which increases their bioactivity. Interconnected pores can provide a framework for bone growth within an implant matrix, holding the prosthesis firmly around the bone and preventing the implant from loosening. These pores can also act as vascular channels that can distribute blood and bone nutrients. One of the most important factors in the bioactivity of ceramics is surface area, which directly determines the solubility rate of each solid. The mechanical properties of porous glasses can be enhanced, and therefore composites of these glasses are made with materials of higher fracture toughness, such as polymers [19], and moreover, with composites whose mineral part is composed of HAp [20]. Bioglass products are best used as a reinforcing material or as a component for making nanocomposites for various applications. HAp porous surfaces provide a suitable template for fibrovascular growth and ectoblast differentiation, thereby preventing new lamellar bone formation. Therefore, the development of high-porosity bone replacements is necessary [21]. The aim of this study was to fabricate HAp cement reinforced with BG in order to improve the compressive strength of HAp cement in SBF solution. The effect of BG on the setting behavior of the cement and the concentration of the liquid phase for cement production was also investigated. In order to review the bioactivity of HAp/BG composite samples in SBF, bioactive tests were conducted to investigate their functionality in physiologic environments and also to investigate the effect of BG nanoparticles on their bioactivity.

## 2. Material and Methods

### 2.1. Fabrication of HAp and Its Composite Cements

HAp and HAp/BG powders were synthesized based on our previous papers [22,23]. Briefly:

HAp nano-powder was synthesized by the hydrothermal method using Ca(NO_3_)_2_·4H_2_O and (NH_4_)_2_HPO_4_ as starting materials, with stoichiometric ratio Ca/P = 1.67. NH_3_ was applied in order to adjust the pH of the solution to a value of 10. The HAp suspension was hydrothermally treated at 130 °C for 10 h.

To synthesize 77s bioglass, glass samples were prepared by the acid catalyzed sol-gel method assisted by the hydrothermal process. Chemical composition by weight was 80 mol% SiO_2_:14 mol% CaO:6 mol% P_2_O_5_.

The hydrothermal method was used for the synthesis of the HAp/BG composite. HAp was mixed with different ratios of glass (i.e., 77s (SiO_2_, CaO, P_2_O_5_)) at a hydrothermal temperature of 180 °C in alkaline condition (pH = 10) for 10 h. Then, the composite powder was dried and washed with distilled water and sintered in a furnace at 700 °C for 2 h.

**Figure 1:**
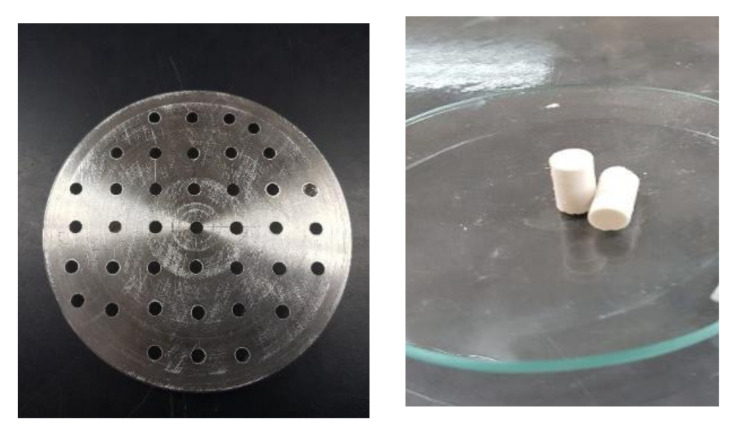
Stainless-steel mold and cement (6 mm diameter and 12 mm height).

To prepare HAp/BG composite cement, synthesized HAp/BG composite powder was mixed with sodium hydrogen phosphate (Na_2_HPO_4_) as the liquid phase in a bowl with a spatula for 30 s. The liquid to powder ratio was 0.40 mL/g. The cement paste was packed into a cylindrical stainless-steel mold (6 mm in diameter and 12 mm in height) to measure compressive strength, and was then pressed using a press machine. The cement was sintered in a furnace at 700 °C for 2 h (Figure 1).

### 2.2. Density and Porosity

The density of cylindrical samples ($\rho_{1}$) was calculated based on previous studies [24], as in Equation (1). The weight of cements after drying (m) and their dimensions (v) were calculated:

(1) $$\rho_{1}=\frac{m}{v}=\frac{m}{h\pi r^{2}}$$

where volume = ${\pi r}^{2}h$, h is the height of the sample, and r is the radius. The relative density (RD) was calculated using Equation (2):

(2) $$RD=\left(\rho_{1}/\rho_{o}\right)\times 100$$

where the density of HAp (reference, $\rho_{0}$) is 3.16 g/cm^3^ [24].

The porosity of cement samples was calculated using the Archimedes method. An average of 5 experiments were used to measure the porosity of the samples. In the first stage, the samples were dried in an oven for 10 h at 100 °C, then dry weight was measured with a digit balance ± 0.001 and labeled as m_dry_, and the samples were suspended in ethanol to fill any pores and their weight was measured and labeled as m_susp_. Then, the ethanol was removed from the samples and this saturation sample was weighted and labeled as m_sat_. The porosity (Φ) of the samples was calculated using Equation (3) [25,26]. The results were expressed as mean ± SD, n = 5.

(3) $$\Phi =\frac{V_{\mathrm{void}}}{V_{\mathrm{total}}}\times 100$$

where the void volume = m_sat_–m_dry_ and total volume = m_sat_–m_susp_.

### 2.3. Setting Time and Compressive Strength Test for Cement

The initial and final setting times of the fabricated cement were measured via Vicat needle apparatus according to the ISO 6876 2001 standard at room temperature. Setting was taken to occur when the Gillmore needle did not impact the sample surface after vertical penetration for between 30 and 60 s. A light needle (8.22 g) with a diameter of 2 mm was used for the determination of the initial time (I) and a heavy needle (30.30 g) with a small diameter (1 mm) was used for the final setting time (F), and the results were expressed as mean ± SD, n = 3. The samples were then sintered for 2 h in a furnace at 700 °C.

**Figure 2:**
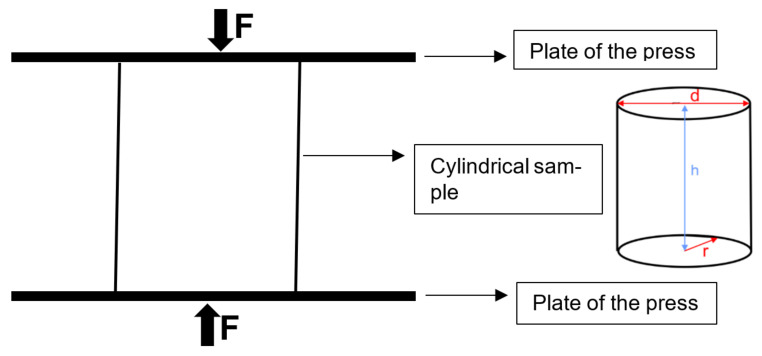
Compressive strength test.

Subsequently, the cement was kept in SBF solution in an incubator for 14 days at 37 °C with a weight-to-volume ratio of 1.5 mg/mL [27]. The compressive strength of the hydroxyapatite and composite cement was measured using an Instron Universal Testing Machine (GOTECH, A1-700LA 10, Taiwan) with a cross-head speed of 1 mm/min (Figure 2). All tests were performed on at least 3 specimens, and the results were expressed as mean ± SD, n = 3. The following equation was used to measure compressive strength [26]:

(4) $$C_{s}=F/A$$

where F is the maximum load (N), A is the cross-sectional surface area (${\pi r}^{2})$ of the sample (mm^2^), and C_s_ is compressive strength. In Figure 2, r is radius, d is diameter, and h is height.

### 2.4. Preparation of Simulated Body Fluid

Simulated body fluid (SBF) solution was prepared based on the Kokubo guidelines under stirrer by dissolving the reagents (i.e., sodium chloride salts, sodium hydrogen carbonate, potassium chloride, di-potassium hydrogen phosphate triplets, and magnesium chloride 6 hydrate) one by one in 1 L of distilled water. Reagent concentrations were as per Table 1. The temperature of the solution was adjusted to 36.5–37 °C with a water bath, and the pH of the SBF solution was kept at 7.4 by using Tris (i.e., hydroxymethyl aminomethane) and HCl solution.

The phase composition of powders was determined using X-ray diffraction (XRD) using a Philips X’pert Pro. The XRD data at room temperature were collected over the 2θ range 10–80°, with a step size of 0.02° and time per scan of 1 s using Cu-Ka radiation (1.5418 A°). Transmission electron microscopy (G2 Spirit BioTWIIN, FEL, USA) was employed to estimate the particle size of the composite powder. An SEM (Hitachi S3400 N, Tokyo, Japan) equipped with an EDX (Quantex200, Bruker, Germany) instrument was used to study the morphology and chemical composition of the samples.

### 2.5. Statistical Analysis

Statistical analysis was carried out using one-way and two-way analysis of variance (ANOVA). Tukey’s test was used to determine the significance of the deviation in the setting time and compressive strength tests of specimens. *p* < 0.05 was considered as a significant level.

## 3. Results and Discussion

**Figure 3:**
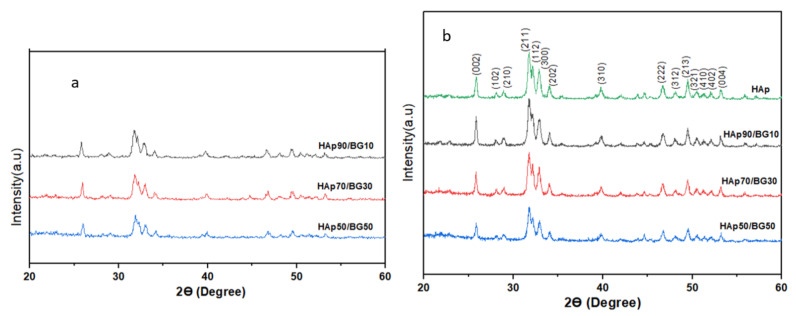
XRD patterns for HAp and HAp/BG cement samples: (**a**) before sintering, and (**b**) after sintering at 700 °C.

Figure 3 shows the XRD patterns of the synthesized HAp and HAp/BG powders before sintering (Figure 3a) and after sintering at 700 °C (Figure 3b). From Figure 3, after sintering the HAp/BG powder at 700 °C, the peaks belonging to HAp became sharper. The sharp peaks indicate the formation of the HAp phase with high crystallinity and no secondary phase (Figure 3b). The peaks were reflected to 2θ = 25.90° (002), 2θ = 31.725° (211), 2θ = 32.18° (112), and 2θ = 32.86° (300), and were found to match well with ICDD 9-432 [28,29]. In Figure 3b, the poor crystallinity in the HAp/BG composites was due to the substitution of Si ions in the HAp structure, which limited the crystallization of the HAp phase. This suggested that the incorporation of BG in the HAp structure decreased the crystallinity of HAp [30].

**Figure 4:**
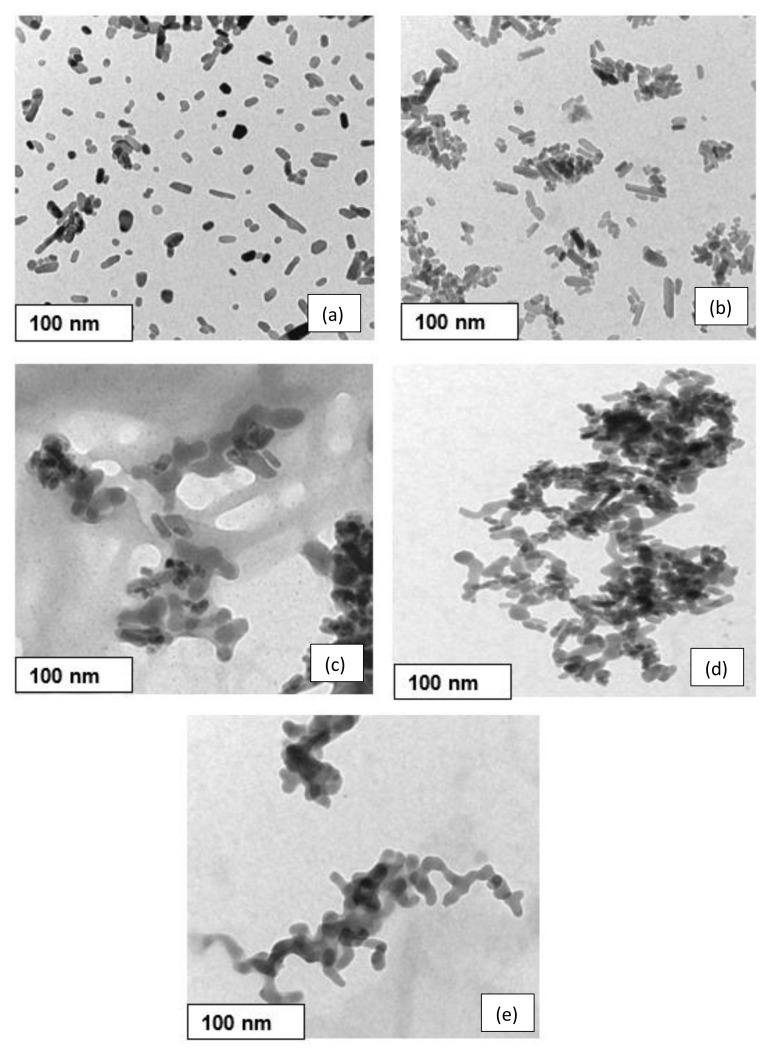
TEM images of composite powders; (**a**) HAp, (**b**) HAp90/BG10, (**c**) HAp70/BG30, (**d**) HAp50/BG50, and (**e**) BG.

Figure 4 and Table 2 show the TEM images and particle size by image analysis (Image J software, version 1.5.3) obtained of prepared HAp, BG, and HAp/BG composite powders under hydrothermal conditions at 180 °C, for 10 h. It is worth mentioning that with the increase of Si content in Si-HAp, the HAp particle size decreased (Table 2). The present results are in agreement with those previously reported by [31,32]. In the study by [31], it was found that by increasing Si content in the HAp/BG composite, the HAp surface was covered by amorphous phase (i.e., BG). Consequently, the thickness of the amorphous phase increased, and Si acted as a barrier to the growth of HAp particles and decreased their size [31].

### 3.1. Density and Porosity Results

Table 3 shows the addition of BG into the HAp with the density and porosity of the composite samples. The results indicated that increasing the amount of BG in the composite reduces the density (i.e., also relative density) and increases the percentage of porosity of the composite samples. This finding is in line with the expectation whereby at higher density, the particles are packed together, resulting in less porosity and vice versa. Therefore, the obtained composite scaffolding largely has the characteristics of different percentages of porosity that relate to its density as a suitable or desirable scaffold used in bone tissue engineering. It is worth mentioning that in this study, with the increase of glass to HAp structure, the density has decreased, and the amount of porosity has increased. Theoretically, the density of glass (2.56 g/cm^3^) is lower than the density of HAp (3.16 g/cm^3^). These results are in good agreement with previous studies [32,33].

**Figure 5:**
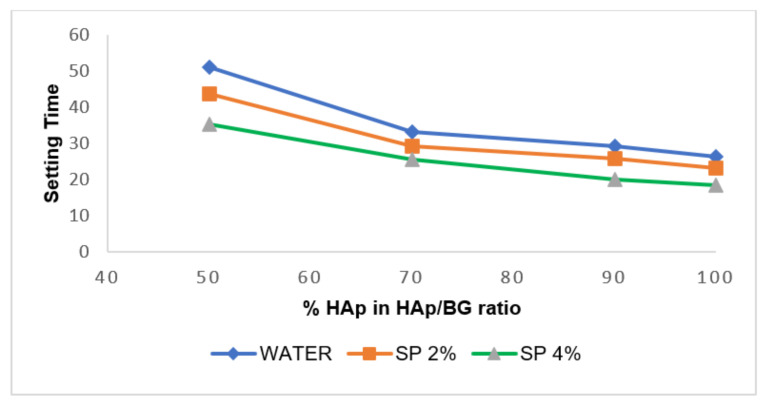
Effect of liquid phase on setting time by HAp percentage in HAp/BG cements (mean ± SD, n = 3, *p* ≤ 0.05).

Table 4 and Figure 5 show the initial and final setting times of the HAp and HAp/BG cements in constant liquid to powder ratio (L/P = 0.4 [34]) at three different components’ weight ratios. It should be mentioned that the ratio of the powders used for manufacturing types of cement (presented in Table 4) is in accordance with the HAp/BG ratio used for synthesizing powders.

Calcium phosphate cement requires time for it to be strong enough to resist an applied force [6]. Therefore, bio cement has optimal clinical parameters, such as initial setting time and final setting time. For this experiment, the setting time of cement was measured for 12 selected samples (i.e., HAp and HAp/BG with different ratios, solvents, and percentages of solvent).

In clinical applications, variables such as initial setting time, final setting time, and strength of the cement are important [35,36].

#### 3.1.1. Effect of Type of Liquid Phase on Setting Time of Cement

In this study, sodium hydrogen phosphate (SP) and water (0% SP) were used as the liquid phases for the production of HAp and HAp cements, and the effect of the liquid phases on the setting time and compressive strength of cement was investigated. From Table 4 and Figure 5, both the initial and final setting times of the cement depended on the type of liquid. By using Na_2_HPO_4_ as the liquid phase, both initial and final setting times decreased, leading to an increased rate of cement formation. However, by using water as the liquid phase, cement production was delayed, and the initial and final setting times of cement were prolonged to 20 and 80 min, respectively. This suggested that the type of liquid phase is crucial because it determines many properties of the cement, such as setting time. A similar finding was also reported by [37], who observed that when distilled water was used as a liquid phase, the setting time of cement was longer than when Na_2_HPO_4_ was used.

#### 3.1.2. Effect of Liquid Phase Concentration on Setting Time of Cement

The concentration of the liquid phase is another factor affecting the setting time of cement. In this study, three different concentrations of sodium hydrogen phosphate were used. As see from Table 4 and Figure 5, the setting time of cement decreases with increased concentration (0% to 4%) of the Na_2_HPO_4_ liquid phase. These results are consistent with previous studies [5] which reported that the function of the liquid phase is to transfer PO_4_^2−^ ions to the reaction medium. This provides suitable super-saturation, thereby accelerating the reaction deposition rate and resulting in decreased setting time for the cement.

#### 3.1.3. Effect of HAp/BG Ratio on Setting Time of Cement

Table 4 shows that the addition of BG into the HAp/BG composite increases the cement setting time. With a constant liquid phase concentration of 2%, an increase in BG from 0% to 50% extended the cement setting time from 23.1 to 43.5 min. This concentration provides more time for surgeons and dentists to perform their tasks before the cement is set. These results demonstrate that the addition of BG raises the composite cement setting time relative to pure HAp cement. [38] reported that an increase in BG concentration to 20% in CPC-BG composites increased the composite cement setting time from 10 to 25 min relative to pure calcium phosphate cement, leading to a homogenous, compact microstructure.

**Figure 6:**
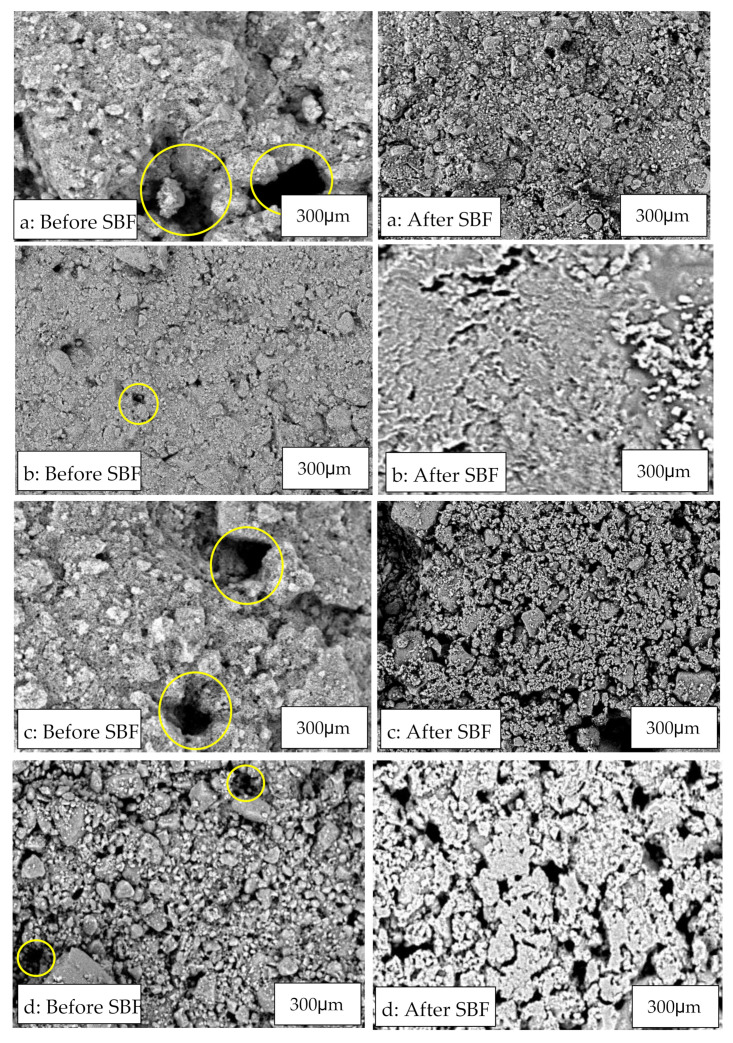
SEM micrographs of cross-sections of HAp/BG composite and HAp samples after hardening: (**a**) HAp50/BG50, (**b**) HAp70/BG30, (**c**) HAp90/BG10, and (**d**) HAp scaffolds. Yellow areas indicate holes in cross-sections of cement.

The SEM images of the HAp cement and HAp/BG composite surfaces indicate that the HAp70/BG30 specimens were combined (Figure 6), suggesting higher microstructural compaction than other types of cement (i.e., HAp90/BG10, HAp50/BG50). However, HAp50/BG50 had numerous pores and lower compaction, leading to a decrease in compressive strength.

### 3.2. Measurement of Compressive Strength

Compressive tests were conducted to determine the effect of BG and to review the function of the production process in improving the mechanical properties of HAp and HAp/BG bioceramic scaffolds. It was expected that the composite scaffolds would show better mechanical behavior than pure apatite. Equation (4) was used to measure compressive strength.

Table 5 shows the compressive strength of cement samples after 14 days in an incubator at 36.5–37 °C (equal to body temperature). The highest compressive strength found was 4.49 MPa for the HAp70/BG30 composite. The factors affecting the compressive strength of cement include the type of liquid phase, its concentration, and the addition of BG to the HAp phase. Table 5 indicates that the concentration of the liquid phase strongly affects the strengthening of the synthesized cement.

#### 3.2.1. Effect of Liquid Concentration on Compressive Strength of HAp and HAp/BG Composite Cement

**Figure 7:**
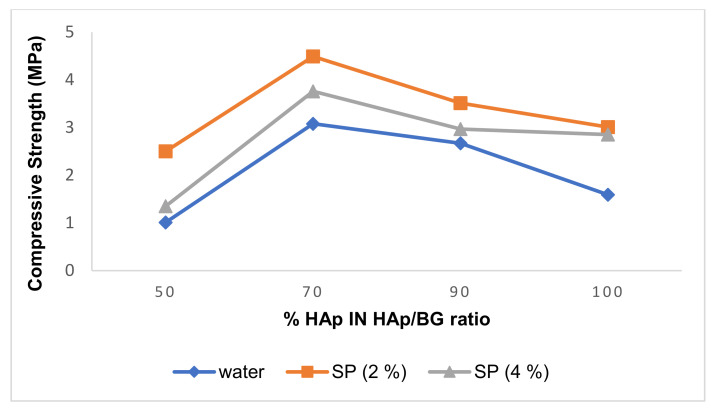
Effect of liquid concentration on compressive strength in HAp and HAp/BG by composite cement percentage.

Table 5 and Figure 7 indicate that the maximum compressive strength is achieved using 2% of SP concentration, although as seen from Table 4, the cement setting time for this concentration is more than for the 4% concentration. Thus, when cement setting time is high, molecules have more opportunities for suitable packing, thus increasing compressive strength. This suggests that the liquid phase concentration is an important parameter for the compressive strength of cement.

The setting time of calcium phosphate cement depends on the kinetics of HAp formation and the diffusion of ions required for its formation. The addition of sodium hydrogen phosphate (SP) as a liquid phase to form HAp cement increases the diffusion of phosphate ions and the kinetics of HAp formation, while reducing the setting time of cement. This increase in setting time increases the compressive strength [39]. As seen from Table 5, the compressive strength of the samples decreased with greater liquid phase concentration. Similar results were obtained in the study by [34]. They reported that the dissolution of powder compounds occurred more quickly at high concentrations of the liquid phase, and the system did not have the necessary time to recrystallize, resulting in a decrease in compressive strength [34].

#### 3.2.2. Effect of HAp/BG Ratio on Compressive Strength of HAp and HAp/BG Cement

**Figure 8:**
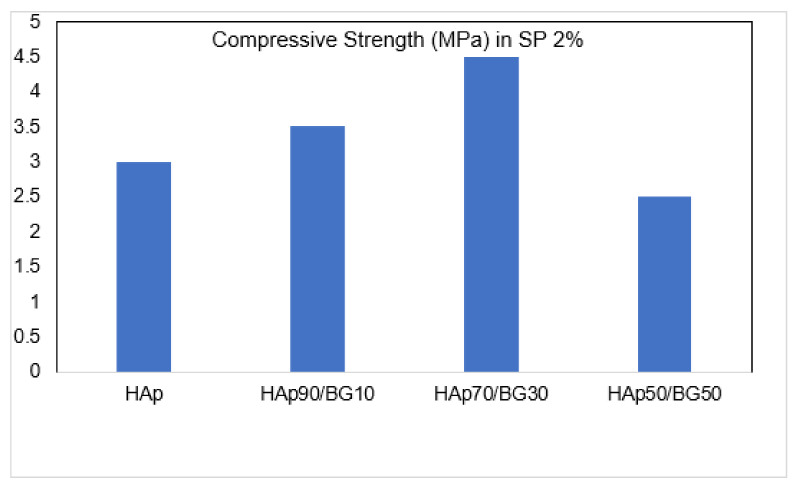
Effect of HAp/BG ratio on compressive strength of cements with SP concentration of 2% (mean ± SD; n = 3, *p* < 0.05).

From Table 5 and Figure 8, it can be seen that increasing BG concentration from 30% to 50% decreases the compressive strength of scaffolds (mean ± SD; n = 3, *p* < 0.05). This suggests that the improved mechanical properties of the HAp/BG composite scaffold (compared to the pure apatite scaffold) can be attributed to the effect of BG as a strengthening phase, and that the mechanical properties of the composite depend on the weight ratio between HAp and BG. The 50% HAp has lower compressive strength than pure Hap, but both 70% and 90% HAp exceed pure HAp. The maximum compressive strength is found in HAp70/BG30.

Figure 6 shows the presence of good packing in the HAp70/BG30 scaffold. In this optimal composition, HAp is packed regularly and is present on the BG surface, increasing mechanical strength. It can be seen that the bioactive glass in the HAp/BG composite becomes smaller and denser after porosity sintering. HAp that is smaller than bioactive glass penetrates into its pores, compacting with bioactive glass, and increasing the compressive strength of the composite. This increase of compressive strength improves the load-bearing capacity of the composite and leads to implant stability in tissue [40].

The maximum force that the samples can endure is increased by raising the concentration of BG, thereby increasing the mechanical strength and leading to the fracture of the samples at higher stresses [33]. The increase of compressive strength of HAp is demonstrated by an increase in the BG phase rate [33]. In other research conducted by [41], it was reported that a 50% increase of BG phase reduces mechanical strength. It seems that by reducing the mechanical properties of HAp/BG scaffolds with increased BG, the glass contains most of the composite. Since the strength of glass is less than that of HAp, the strength of the composite is decreased. With more glass in the composite, leading to increased pores in the nanostructure, silicated HAp formation is reduced [33,42]. In addition, increasing the BG phase from 30 to 50 wt.% reduces the density, compared to pure HAp. Therefore, the HAp/BG with 50 wt.% was dissolved in SBF solution [43].

Considering the two key parameters of setting time and compressive strength of the synthesized bone cement, the cement with a liquid phase concentration of 2% and a HAp70/BG30 ratio with a liquid to solid phase ratio of 0.4 can be selected for the production of HAp and HAp/BG cement with sintering at 700 °C for 2 h.

### 3.3. In Vitro Bioactivity of the Composite Cements

Figure 6 shows SEM images of scaffold samples before and after immersion in SBF solution at 37 °C for 14 days. These images demonstrate improved formation of apatite crystals on the surface of porous scaffolds, with HAp70/BG30 achieving complete coverage (Figure 6b). The surface of pure HAp is also mostly covered by an apatite layer (Figure 6d). HAp50/BG50 and HAp90/BG10 surfaces were only partially covered by an apatite layer (Figure 6a,c, respectively). As indicated by the SEM images, the presence of BG nanoparticles in the HAp/BG scaffolds leads to increased bioactivity, which can be attributed to the effect of groups (SiOH_4_) in the stimulation of the nucleation process and the growth of apatite crystals [38]. The release of Ca^2+^ and PO_4_^2−^ ions from HAp/BG porous scaffolds increases super-saturation of these ions in the SBF solution. By exchanging Ca^2+^ groups with H_3_O^+^ in SBF and continuing the formation of (Si-OH) groups on the surface, nucleation and the growth of HAp crystals increased. By raising BG concentration, silanol groups are formed due to the interactions between the surface and the SBF thereon, thus increasing nucleation and the growth of HAp crystals [30]. Comparing SEM images of HAp/BG (Figure 6b) nanocomposites with pure HAp (Figure 6d), a considerable increase in bioactivity for the nanocomposite over HAp alone can be seen. Similar results have been obtained in preparing other composites composed of HAp and bioactive glass [44]. In another study conducted by [30], the bioactivity of HAp and Si-HAp in SBF solution was evaluated at 3-, 8-, and 14-day intervals. The results of that study showed that the density of apatite crystals on the Si-HAp surface exceeded that of HAp after 3 days, and that some parts of the Si-HAp were fully covered by apatite layers after 8 days. In contrast, significant changes on the HAp surface (i.e., surface covering by apatite layers) were observed only after 14 days. This study showed that the Si-HAp biological activity is more than that of HAp [45].

**Figure 9:**
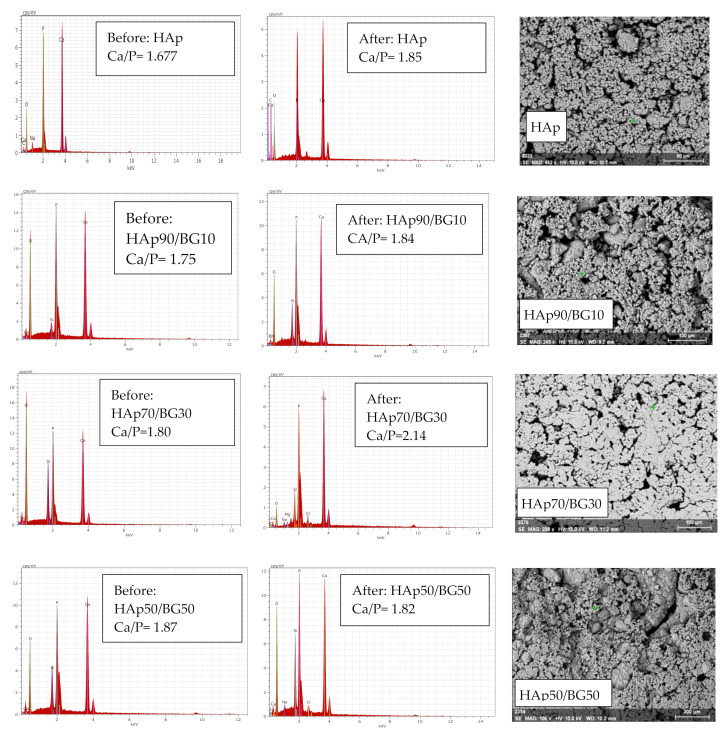
SEM images after immersion and EDX results for samples before and after 14 days of immersion in SBF solution.

To confirm the formation of an apatite layer on the surface of HAp and HAp/BG cement after immersion into SBF solution, we utilized SEM/EDX analysis (Figure 9). Figure 9 and Table 6 show the EDX results after immersion of samples into SBF solution. From the EDX results, the elements P, Ca, Si, O, and some Na, Cl, and Mg ions were detected after immersion for 14 days. The percentages of Ca and P increased after soaking in the SBF solution, which indicates the formation of an apatite layer on the surface of the cement. In the sample HAp50/BG50 (for which SEM results showed to be partially covered with apatite), the Ca/P ratio was found to decrease. This may be because of the sample’s highly amorphous structure and the high solubility and, in fact, the scaffold surface in the SBF solution was a little brittle and cracked, which decreased the density of the apatite layer and resulted in the lowest Ca/P ratio and the lowest bioactivity. Additionally, the results of immersion into SBF solution revealed that most changes in the Ca/P ratio were found for the HAp70/BG30 sample, indicating the highest bioactivity of all samples. These results agree with previous research [46,47].

## 4. Conclusions

In this work, HAp/BG composite cement was fabricated and then characterized. Setting times of the cement at different concentrations of liquid phase sodium hydrogen phosphate (Na_2_HPO4) (i.e., SP 0%, 2%, and 4%) were found to decrease with a greater concentration of the liquid phase, but were prolonged with increased content of BG in the composite. Furthermore, incorporation of BG into the HAp structure appeared to decrease the density and to increase the porosity. It was also found that the compressive strength of the HAp and HAp/BG cements after immersion in SBF solution was increased by increasing the amount of BG phase up to 30 wt.%. In addition, the presence of BG nanoparticles in the HAp/BG scaffolds resulted in significantly improved bioactivity. Therefore, the incorporation of BG into HAp appears to yield a suitable scaffold for clinical applications.

## Figures and Tables

**Table 1:** Ion concentrations of prepared SBF (simulated body fluid) and human blood plasma (1 L).

Order	Reagent	Ion	Amount (g)	Concentration (m Mol·dm^−3^)	
				(SBF)	Human Blood Plasma
1	NaCl	Na^+^	7.996	142.0	142.0
2	KCl	K^+^	0.224	5.0	5.0
3	MgCL_2_·6H_2_O	Mg^2+^	0.305	1.5	1.5
4	CaCl_2_	Ca^2+^	0.278	2.5	2.5
5	HCl (1 M)	Cl^−^	40 mL	147.8	103.0
6	NaHCO_3_	HCO^3−^	0.350	4.2	27.0
7	K_2_HPO_4_·3H_2_O	HPO_4_^2−^	0.228	1.0	1.0
8	Na_2_SO_4_	SO_4_^2−^	0.071	0.5	0.5
9	(CH_2_OH)_3_CNH_2_ (Tris)	-	6.057	-	-

**Table 2:** Particle size obtained from TEM images by Image J software.

HAp	HAp90/BG10	HAp70/BG30	HAp50/BG50	BG
29.53 ± 5.37	25.60 ± 8.30	22.82 ± 8.11	20.46 ± 6.73	79.017 ± 50.132

**Table 3:** Addition of BG in the HAp/BG composite with the density, relative density (RD), and porosity percentage (the results were expressed as mean ± SD, n = 5).

Sample	Density ($\rho_{1}$, g/cm^3^)	RD (%)	Porosity (Φ, %)
HAp	1.88 ± 0.56	59	39 ± 1.44
HAp90/BG10	1.71 ± 0.31	54	41 ± 1
HAp70/BG30	1.59 ± 0.58	50	48 ± 3.5
HAp50/BG50	1.41 ± 0.24	44	55 ± 2.23

**Table 4:** Setting times of HAp and HAp/BG cements prepared using different weight ratios (the results were expressed as mean ± SD, n = 3).

Powder Phase		Liquid Phase	Initial Time	Final Time	Setting Time (ST)
HAp%	BG%	SP (0% i.e., water)	20.21 ± 0.8	46.39 ± 0.24	26.18
100	0	SP (2%)	19.32 ± 0.75	42.42 ± 0.68	23.1
100	0	SP (4%)	13.99 ± 0.34	32.24 ± 0.89	18.25
100	0	SP (0% i.e., water)	24.97 ± 0.65	54.21 ± 1.23	29.24
90	10	SP (2%)	21.51 ± 0.42	46.94 ± 0.84	25.43
90	10	SP (4%)	16.26 ± 0.94	36.29 ± 0.56	20.03
90	10	SP (0% i.e., water)	34.37 ± 1.1	67.44 ± 0.79	33.07
70	30	SP (2%)	27.29 ± 0.68	56.49 ± 0.84	29.2
70	30	SP (4%)	24.85 ± 0.48	50.25 ± 1.45	25.4
70	30	SP (0% i.e., water)	36.31 ± 0.87	87.18 ± 1.89	50.87
50	50	SP (2%)	30.27 ± 0.75	73.77 ± 0.89	43.5
50	50	SP (4%)	27.5 ± 0.59	62.75 ± 1.14	35.25

**Table 5:** Effects of liquid phase concentration on compressive strength (the results were expressed as mean ± SD, n = 3).

Powder Phase	Liquid Phase	Compressive Strength (MPa)
HAp	SP (0% i.e., water)	1.59 ± 0.41
HAp	SP (0.02%)	3.01 ± 0.15
HAp	SP (0.04%)	2.85 ± 0.17
HAp90/BG10	SP (0% i.e., water)	2.67 ± 0.18
HAp90/BG10	SP (0.02%)	3.51 ± 0.29
HAp90/BG10	SP (0.04%)	2.97 ± 0.37
HAp70/BG30	SP (0% i.e., water)	3.08 +0.40
HAp70/BG30	SP (0.02%)	4.49 ± 0.25
HAp70/BG30	SP (0.04%)	3.76 ± 0.18
HAp50/BG50	SP (0% i.e., water)	1.01 ± 0.21
HAp50/BG50	SP (0.02%)	2.50 ± 0.17
HAp50/BG50	SP (0.04%)	1.35 ± 0.15

**Table 6:** Atomic composition of samples of HAp and HAp/BG composites before and after immersion in SBF solution, from EDX analysis (n = 4).

Element	HAp		HAp90/BG10		HAp70/BG30		HAp50/BG50	
Atomic %	Before SBF	After SBF	Before SBF	After SBF	Before SBF	After SBF	Before SBF	After SBF
O	60.47	46.51	68.94	58.15	72.85	37.50	61.79	64.51
Ca	16.81	27.05	19.05	21.46	14.40	38.23	22.01	18.74
P	10.02	14.62	10.85	11.75	7.99	17.82	11.77	10.25
Si	0	0	0.87	3.08	4.76	4.20	4.43	5.34

## Data Availability

Data is contained within the article.
